# Effectiveness, Usability, and Acceptability of ChatGPT With Retrieval-Augmented Generation (SIV-ChatGPT) in Increasing Seasonal Influenza Vaccination Uptake Among Older Adults: Quasi-Experimental Study

**DOI:** 10.2196/76849

**Published:** 2025-09-08

**Authors:** Zixin Wang, Tsz Hin Tsang, Fuk-yuen Yu, Yuan Fang, Siyu Chen, Fenghua Sun, Phoenix K H Mo, Kwong-Cheong Wong

**Affiliations:** 1 Centre for Health Behaviours Research, JC School of Public Health and Primary Care, The Chinese University of Hong Kong Hong Kong China (Hong Kong); 2 School of Governance and Policy Science, The Chinese University of Hong Kong Hong Kong China (Hong Kong); 3 Department of Health and Physical Education, The Education University of Hong Kong Hong Kong China (Hong Kong)

**Keywords:** ChatGPT, retrieval-augmented generation, health promotion, seasonal influenza vaccination, older adults, quasi-experimental study

## Abstract

**Background:**

Older adults are more vulnerable to severe consequences caused by seasonal influenza. Although seasonal influenza vaccination (SIV) is effective and free vaccines are available, the SIV uptake rate remained inadequate among people aged 65 years or older in Hong Kong, China. There was a lack of studies evaluating ChatGPT in promoting vaccination uptake among older adults.

**Objective:**

This study aimed to evaluate the effectiveness of ChatGPT with retrieval-augmented generation in increasing SIV uptake among older adults over a 3-month study period in Hong Kong, China. Participants in an ongoing observational cohort study conducted in the same period served as the comparison group.

**Methods:**

A quasi-experimental study was conducted between November 2024 and April 2025. Participants were (1) aged ≥65 years, (2) possessed a Hong Kong ID, (3) able to speak and comprehend Cantonese, (4) smartphone users, and (5) had no SIV uptake for the approaching flu season. Those with a diagnosis of cognitive impairment or dementia, blindness or deafness, or known contraindications to the SIV were excluded. Participants were recruited through random telephone calls. There were 45 and 55 participants in the SIV-ChatGPT group and the comparison group, respectively. All participants completed follow-up surveys at T1 (1 month after the baseline survey, for the SIV-ChatGPT group only) and T2 (3 months after the baseline survey, for both groups). Participants in the SIV-ChatGPT group gained access to SIV-ChatGPT in the format of a web-based app after completion of the baseline survey. They could use SIV-ChatGPT repeatedly throughout a 1-month intervention period and were free to spend as much time as they wanted with SIV-ChatGPT. Intention-to-treat analysis was used for outcome analyses.

**Results:**

At T2, the SIV uptake rate was higher in the SIV-ChatGPT group than the comparison group (15/45, 33% vs 8/55, 14.3%; adjusted odds ratio 2.72, 95% CI 1.01-7.35, *P*=.048). All participants were able to provide receipts to validate their SIV uptake. In the SIV-ChatGPT group, 40.5% (15/37) of participants who used SIV-ChatGPT at least once reported a SIV uptake at T2, which was significantly higher than nonusers (0/8, 0%; *P*=.04). Among the 37 SIV-ChatGPT users, the mean score of the System Usability Scale was 67.1 (SD 14.9). Levels of subjective behavioral and cognitive engagement with SIV-ChatGPT were relatively high, while the affective engagement was moderate.

**Conclusions:**

SIV-ChatGPT was feasible and acceptable and demonstrated preliminary effectiveness in increasing SIV uptake among people aged 65 years or older. This study also provided implications to improve the performance of SIV-ChatGPT. A full-powered randomized controlled trial should be considered to evaluate its efficacy.

**Trial Registration:**

ClinicalTrials.gov NCT06679647; https://clinicaltrials.gov/study/NCT06679647

## Introduction

There are 2 flu seasons in Hong Kong (January-March and July-August). In the 2023/24 flu season, people aged ≥65 years accounted for 89% of the deaths caused by seasonal influenza [1]. The seasonal influenza vaccination (SIV) could effectively prevent influenza and its complications among people aged 65 years or older without safety concerns [2,3]. All Hong Kong residents of this age group are strongly recommended to receive an SIV annually [4]. Hong Kong residents of this age group can receive either free SIV at public hospitals or subsidized vaccines at private clinics [5]. However, the SIV coverage in people aged 65 years or older remained inadequate in the past 3 years (40.4%-51.5%) [6]. Room for improvement is conspicuous.

Previous research emphasizes the importance of providing adequate information regarding vaccination. A recent meta-analysis showed that providing information via written messages could increase SIV uptake by 16% compared with no intervention [7]. Chatbots are computerized programs that replicate human interactions through various forms of communication [8], and their popularity is increasing in health promotion. Chatbots can proactively interact with users and provide information that is tailored to users’ characteristics and needs. A meta-analysis and recent randomized controlled trials demonstrated that Chatbots had good abilities to reach the target population and were promising in increasing vaccination uptake [9-11]. Apart from one chatbot targeting parents to promote human papillomavirus vaccination uptake among their daughters [11], other vaccination-promotion chatbots (n=14) identified by our literature search were not based on large-language models (LLMs) and hence had limited ability to interpret and answer users’ free-text questions in real-time [9,10]. These open-ended questions usually reflect users’ important concerns and cannot be fully covered by predefined, frequently asked questions. Some non-LLM chatbots could extract keywords from users’ questions and retrieve predefined answers in real-time [9,10]. However, the question-answer (QA) interaction of these chatbots relies on predefined rules, which raises concerns about the accuracy. Moreover, many users criticized that these QA interactions were more robotic than human-like, and ultimately, such constraints negatively affected users’ engagement with the chatbots [9]. Better engagement with chatbots was associated with higher motivation to receive vaccination [9]. Previous studies have shown that providing real-time and appropriate answers to users’ questions could increase engagement with web-based interventions [12].

ChatGPT is developed with the generative pretrained transformer [13], an autoregressive LLM using deep learning to generate human conversation. As compared with non-LLM-based conventional Chatbots, ChatGPT can better understand users’ questions and generate more well-written and human-like responses. Moreover, ChatGPT can be trained to perform specific tasks. Previous studies invited independent experts to evaluate ChatGPT-generated responses to open-ended questions related to vaccination and found those responses to be accurate, clear, and concise [14]. Retrieval-augmented generation (RAG) is a technique to enhance the accuracy and reliability of ChatGPT with information retrieved from specific and relevant data sources [15]. Previous studies consistently showed that integrating LLM with RAG could largely increase the accuracy of LLM-generated responses to health-related queries; the accuracy rate of the responses could reach 98%-100% [16,17]. Therefore, the application of RAG in this study could prevent hallucination issues [13]. A previous study successfully applied ChatGPT with RAG to encourage parents to have their daughters receive human papillomavirus vaccination [11]. ChatGPT demonstrated preliminary effectiveness in health promotion (ie, reduce loneliness) for older adults [18]. However, there was a lack of studies evaluating the effectiveness, usability, and acceptability of ChatGPT in promoting vaccination uptake among older adults.

To address the knowledge gap, this study invited community-dwelling Hong Kong residents aged 65 years or older to use a ChatGPT with RAG (the SIV-ChatGPT group). Participants of a separate and ongoing observational prospective cohort study conducted during the same period served as the comparison group. This study aimed to examine the effectiveness of SIV-ChatGPT by comparing SIV uptake rate between the SIV-ChatGPT group and the comparison group 3 months after the baseline survey (T2). This study also evaluated the usability and acceptability (extent of usage and subjective experiences of behavioral, cognitive, and affective engagement) of SIV-ChatGPT at the end of the 4-week intervention period (T1). In addition, changes (T1 vs baseline) in behavioral intention to receive SIV and attitudes toward SIV among participants in the SIV-ChatGPT group were investigated. We hypothesized that the SIV uptake rate among participants in the SIV-ChatGPT group would be higher than that of the comparison group at T2.

## Methods

### Study Design

**Figure 1:**
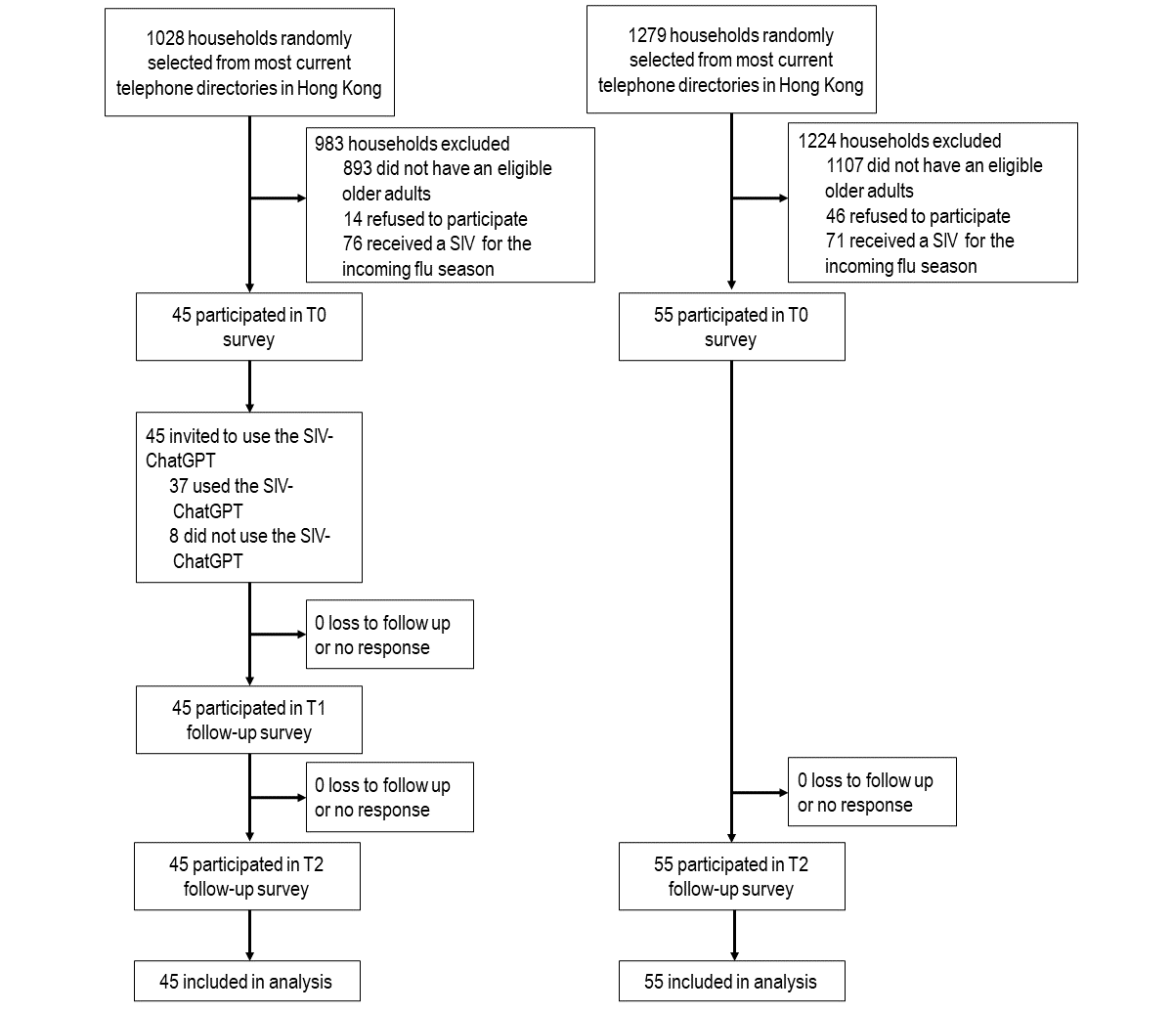
Flowchart diagram of this study. SIV: seasonal influenza vaccination.

A quasi-experimental study was conducted between November 30, 2024 and April 22, 2025. Participants in the SIV-ChatGPT group completed a telephone survey and gained access to SIV-ChatGPT at baseline (T0). They could use SIV-ChatGPT repeatedly throughout a 1-month intervention period and were free to spend as much time as they wanted with SIV-ChatGPT. Two telephone evaluation surveys were conducted after completion of the intervention (T1) and 3 months after T0 (T2). This study was prospectively registered with ClinicalTiral.gov (NCT06679647) on November 7, 2024. In the same city and between November and December 2024, older adults who did not receive SIV for the approaching flu season in an ongoing observational cohort that investigated changes in health behaviors served as the comparison group. The cohort participants completed 2 telephone surveys 3 months apart. The flowchart of this study is shown in Figure 1.

### Ethical Considerations

The Survey and Behavioral Research Ethics Committee of the Chinese University of Hong Kong approved this study (SBRE 24-0263 and SBRE-20-670). The procedures of this study were in accordance to the ethical standards of the ethical standards of the aforementioned responsible committees and with the WMA Declaration of Helsinki.

The interviewers screened prospective participants for eligibility, briefed them about the study, and guaranteed their anonymity and right to quit at any time. Participants were briefed that their chat history with SIV-ChatGPT would be encrypted and would not be used by OpenAI or any third parties. Only the principal investigator of this study had access to the data. The chat history would be removed once the project ended. Participants were asked whether they understood the briefing and whether they were willing to join the study. A hotline was available for inquiries. Since there was no face-to-face contact, verbal informed consent was obtained. The interviewers signed a form pledging that the participants had been fully informed about the study. The recruitment and data collection methods were identical between the SIV-ChatGPT group and the comparison group. An HK $50 (US $6.4) supermarket coupon was given after the completion of each survey.

### Participants and Data Collection

The inclusion criteria of the SIV-ChatGPT group and the comparison group were (1) aged ≥65 years, (2) possession of a Hong Kong ID, (3) able to speak and comprehend Cantonese, (4) smartphone users, and (5) no SIV uptake for the approaching flu season. The exclusion criteria include (1) with diagnosis of cognitive impairment or dementia, (2) either blindness or deafness, (3) with known contraindications to the SIV as indicated by the Hong Kong Centre for Health Protection (eg, history of severe allergic reactions to any of the vaccine component or a previous dose of SIV and bleeding disorders) [4].

We aimed to recruit 50 participants to use SIV-ChatGPT and a similar number of participants in the comparison group. Previous surveys showed that 45% of community-dwelling Hong Kong residents aged 65 years or older intended to receive a SIV for the approaching flu season [19]. Assuming 40% (n=9) of participants in the comparison group with an intention at baseline would take up a SIV within 3 months (18% of 50 participants), such sample size could detect a smallest difference of 25% in SIV uptake between the SIV-ChatGPT group and the comparison group (n=22, 43% vs n=9, 18%; power 0.80, α=.05; PASS 11.0, NCSS). Regarding the within-group changes in outcomes, the sample size of the SIV-ChatGPT group could detect a moderate before and after mean difference among participants (Cohen *d*=0.40) via a paired *t* test, with a power of 80% at an α level of 5% of a 2-tailed significance and a SD of difference at 1.0.

Participants in the SIV-ChatGPT group were recruited through random telephone calls, a method we have used to recruit older adults in Hong Kong in our previous interventional studies [20]. Over 90% of Hong Kong people aged ≥60 years have a fixed-line number [21]. All household telephone numbers listed in the up-to-date telephone directories (about 350,000 records) were input into a spreadsheet file (Excel; Microsoft Corp). About 1000 household fixed-line telephone numbers were randomly selected. Trained telephone interviewers conducted the telephone calls between 6 PM and 10 PM on weekdays and between 2 PM and 9 PM on Saturdays to avoid undersampling of working individuals. If no one in the selected household answered the initial call, 4 more follow-up calls were made on different days and hours before the household was considered as nonvalid. If there was more than one eligible person in the household, the one whose birthday is closest to the interview date would be invited to join the study.

### Development of SIV-ChatGPT

SIV-ChatGPT was supported by a comprehensive QA database. The research team extracted SIV-related information from governmental websites, literature reviews, our previous surveys/interviews with older adults [22], and questions raised by participants of previous SIV promotion programs [20]. A panel consisting of investigators (experts in public health, vaccination behaviors, and health psychology) and 5 older adults held multiple meetings, taking the findings into account for preparing comprehensive QA pairs concerning SIV.

**Figure 2:**
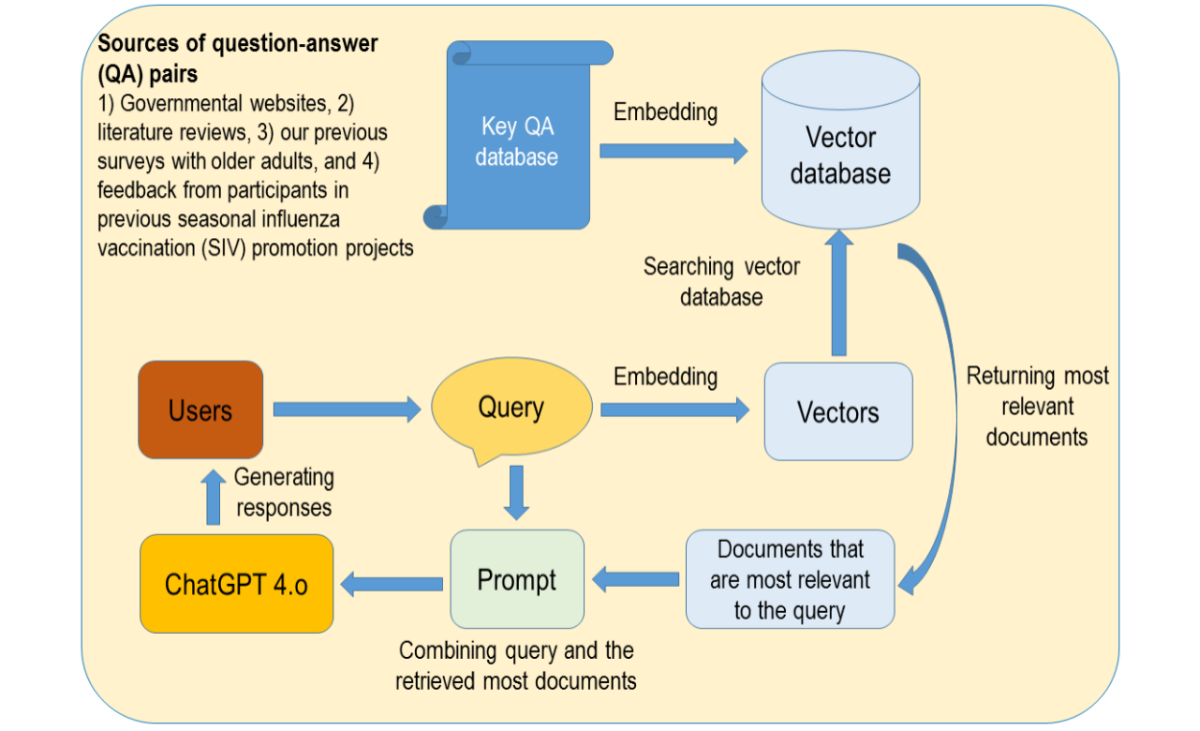
Conversation mechanism of seasonal influenza vaccination (SIV)-ChatGPT. QA: question-answer.

SIV-ChatGPT used the following mechanism to generate responses to users’ queries (Figure 2):

1. The QA database was applied with word embedding that converted data into numerical representations and stored in a vector database.
2. The user’s query was also converted into numerical representations. The system then selected documents from the vector database that were most relevant to the query. The relevancy was calculated by both keyword search and semantic similarity.
3. The system returned these highly relevant retrieved documents that contained the answers to users’ queries. The system then added these highly relevant documents to the user’s query and fed both the documents together with the query into the GPT4o via a prompt.
4. The GPT4o generated responses based on both the user’s query and these highly relevant documents, which ensured that the responses were accurate and appropriate.

**Figure 3:**
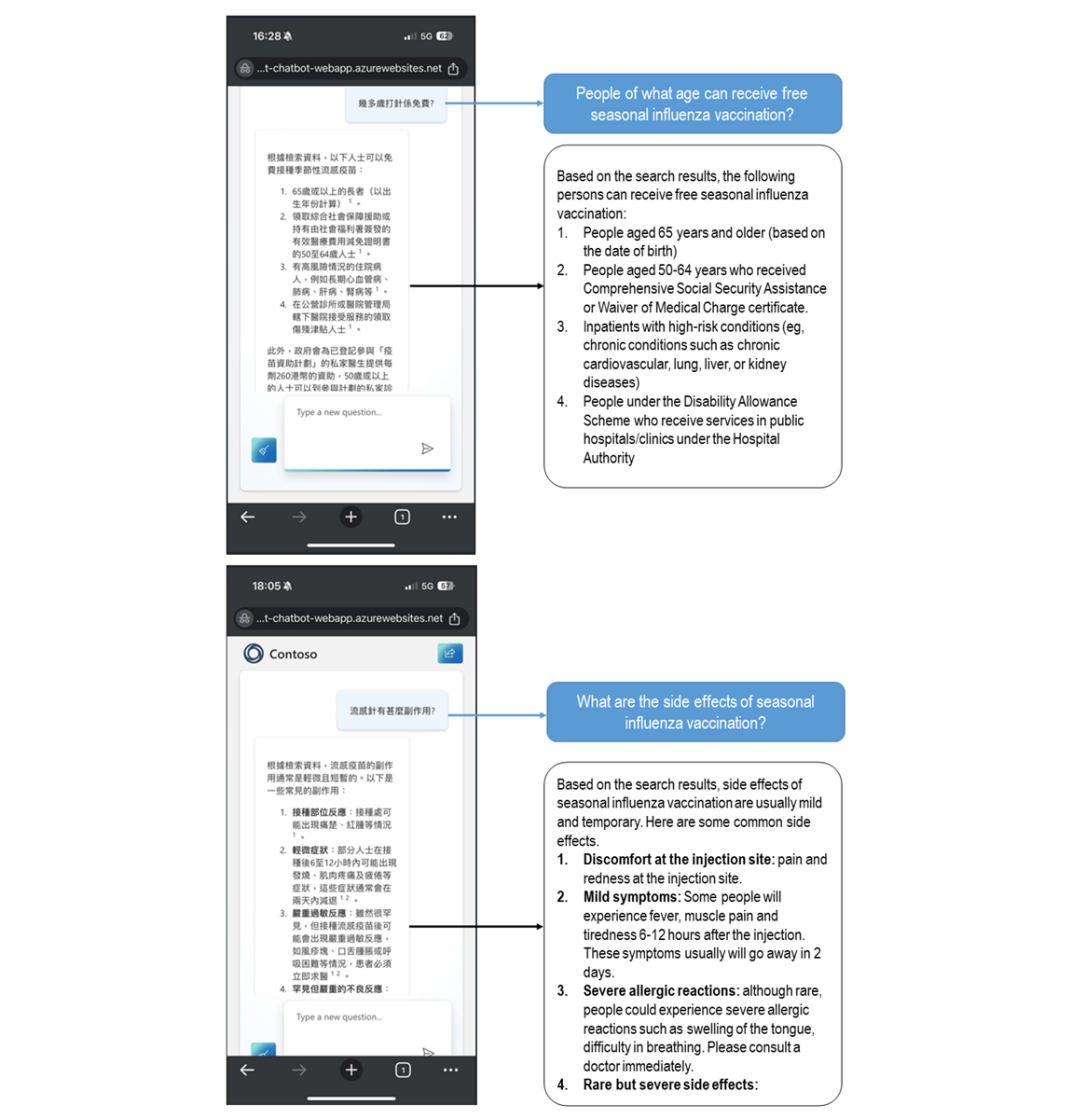
Interface of seasonal influenza vaccination (SIV)-ChatGPT.

Interfaces of SIV-ChatGPT are shown in Figure 3.

Participants’ chat history with SIV-ChatGPT was protected by the OpenAI secure protocol. The data was encrypted on OpenAI server, and would not be used by OpenAI or any third parties. Only the principal investigator had access to the data. The chat history was removed from the server once the project ended.

### Validation of SIV-ChatGPT–Generated Responses

Similar to the approaches used in previous studies [14,23-25], 2 experts in vaccination behaviors independently evaluated SIV-ChatGPT–generated responses to 20 randomly selected open-ended SIV-related questions twice, 1 week apart. We used the 3C criteria for our evaluation (Correct: accurate contents; Clear: clear contents; and Concise: all available knowledge is conveyed succinctly) [14]. A score was given to each criterion for each response (4=completely correct, clear or concise, 3=almost correct, clear or concise, 2=partially correct, clear or concise, and 1=completely incorrect, unclear or unconcise). The mean score was 3.9 (SD 0.3) for correct, clear, and concise, which indicated that the overall contents were correct, clear, and concise to a large degree. The inter-rater reliability indicated good agreement (absolute agreement: 96.7%), and test-retest reliability indicated that the performance of SIV-ChatGPT was stable over time (Cohen κ 0.86).

### The SIV-ChatGPT Group

SIV-ChatGPT, in the format of a web-based app, was not publicly available at this stage. Participants in the SIV-ChatGPT group could access the app through a link/QR code sent by the research team during the intervention period. A unique username and password were assigned to each participant. The participants needed to enter their username and password when they used the app for the first time. Participants were invited to raise open-ended questions related to SIV by typing in traditional Chinese. SIV-ChatGPT answered these questions immediately in the format of text messages. The functions can be used repeatedly throughout the intervention period (1 month), and participants were free to spend as much time as they wanted on such functions.

### The Comparison Group

The research team did not inform participants in the comparison group about SIV-ChatGPT during the study period. Participants in the SIV-ChatGPT group did not overlap with those in the comparison group. It was possible for participants in this group to obtain information about SIV-ChatGPT from elsewhere (eg, their peers) and approach the research team to use SIV-ChatGPT. However, none of them made such a request during the study period.

### Outcome Measurements

We measured self-reported SIV uptake among participants in both the SIV-ChatGPT group (measured at T1 and T2) and the comparison group (measured at T2). Same as previous interventional studies [20,26], the research team validated this outcome by requesting the participants upload an image of their SIV receipt, while hiding their personal identification, via WhatsApp. No incentive will be given for validation. In addition, behavioral intention to receive SIV for the approaching flu season and attitudes toward SIV (ie, perceived risk and severity of influenza infection, perceived benefits, cost, and self-efficacy related to SIV) were measured using validated tools among participants in the SIV-ChatGPT group at T0 and T1 [22].

The following outcomes were collected among participants in the SIV-ChatGPT group at T1, including (1) perceived usability of SIV-ChatGPT assessed by the validated Chinese version of the 10-item System Usability Scale (SUS) [27]; (2) extent of usage documented by the intervention system; (3) subjective experiences related to behavioral (eg, low efforts is required for using the technology), cognitive (eg, perceiving the technology can support and motivate them to achieve their goal), and affective (eg, enjoyment, satisfaction when using the technology) engagement measured by the validated Twente Engagement with eHealth Technologies Scale (TWEETS) [28]; and (4) user’s open-ended comments and suggestions on the use of SIV-ChatGPT. With verbal informed consent, these comments and suggestions were audio-recorded.

### Statistical Analysis

Descriptive statistics were used to characterize the sample and study outcomes. Between-group differences in baseline characteristics were compared using chi-square tests. Since there was no dropout at T2 and no missing data at T1 and T2, there was no need to fill in the missing outcomes. Intention-to-treat analysis was used to test the between-group difference in SIV uptake. Logistic regression model was used to test the between-group difference in SIV uptake at T2, after controlling for baseline background characteristics with *P*<.05 in between-group comparisons. Crude odds ratios (OR), adjusted OR, and their 95% CI were obtained. Changes in behavioral intention and attitudes related to SIV between T1 and T0 in the SIV-ChatGPT group were investigated using McNemar tests (for categorical variables) or paired-sample *t* tests (for continuous variables). Analyses were conducted using SPSS (version 26.0; IBM, SPSS Inc), and *P*<.05 was considered statistically significant. Regarding qualitative feedback of SIV-ChatGPT, the research team transcribed the interviews and kept a code book to record special data and transformed the data into categories to identify main themes.

## Results

### Baseline Characteristics of Participants

From November 30, 2024 to January 14, 2025, 1028 households were contacted to recruit participants of the SIV-ChatGPT group, 59 had an eligible older adult, and 45 completed the baseline survey. Regarding the prospective cohort, 1279 households were called to recruit participants, 172 households had an eligible older adult, and 126 households completed the baseline survey. This study was based on a subsample of 55 participants who did not receive SIV for the approaching flu season (since October 2024) during the recruitment period. At baseline, the majority of the participants were aged 65-69 years (67/100, 67%), female (64/100, 64%), married or cohabiting with a partner (56/100, 56%), and without a tertiary education (82/100, 82%). Among the participants, 46% (n=46) had at least one chronic condition, and 84% (n=84) reported a history of confirmed COVID-19 infection. Less than half of them received SIV in the previous flu season (October 2023 to September 2024) (42/100, 42%). Apart from the history of confirmed SARS-CoV-2 infection (*P*=.02), no significant between-group difference in baseline characteristics was found (Table 1).

**Table 1:** Baseline characteristics of the participants.

				Total (n=100), n (%)		SIV^a^-ChatGPT group (n=45), n (%)	Comparison group (n=55), n (%)	*P* value
**Sociodemographics**								
	**Age group (years)**							
		65-69	67 (67)		28 (62.2)		39 (70.9)	—^b^
		70-74	24 (24)		14 (31.1)		10 (18.2)	—
		≥75	9 (9)		3 (6.7)		6 (10.9)	.29
	**Sex assigned at birth**							
		Male	36 (36)		12 (26.7)		24 (43.6)	—
		Female	64 (64)		33 (73.3)		31 (56.4)	.08
	**Relationship status**							
		Single	44 (44)		17 (37.8)		27 (49.1)	—
		Married or cohabiting with a partner	56 (56)		28 (62.2)		28 (50.9)	.26
	**Education level**							
		Primary or below	19 (19)		7 (15.6)		12 (21.8)	—
		Secondary	63 (63)		29 (64.4)		34 (61.8)	—
		Tertiary or above	18 (18)		9 (20)		9 (16.4)	.70
	**Monthly household income, HK$ (US $)**							
		<20,000 (2580)	73 (73)		32 (71.1)		41 (74.5)	—
		≥20,000 (2580)	24 (24)		12 (26.7)		12 (21.8)	—
		Refuse to disclose	3 (3)		1 (2.2)		2 (3.6)	.80
	**Receiving CSSA^c^**							
		No	97 (97)		42 (93.3)		55 (100)	—
		Yes	3 (3)		3 (6.7)		0 (0)	.09
	**Living alone**							
		No	72 (72)		32 (71.1)		40 (72.7)	—
		Yes	28 (28)		13 (28.9)		15 (27.3)	.86
**Lifestyles and health conditions**								
	**Smoking in the past year**							
		No	97 (97)		44 (97.8)		53 (96.4)	—
		Yes	3 (3)		1 (2.2)		2 (3.6)	.68
	**Binge drinking in the past year**							
		No	97 (97)		43 (95.6)		54 (98.2)	—
		Yes	3 (3)		2 (4.4)		1 (1.8)	.44
	**Presence of some chronic condition, yes**							
		Hypertension	38 (38)		20 (44.4)		18 (32.7)	.23
		Chronic cardiovascular diseases	3 (3)		3 (6.7)		0 (0)	.09
		Chronic lung diseases	1 (1)		0 (0)		1 (1.8)	.36
		Chronic liver diseases	4 (4)		2 (4.4)		2 (3.6)	.84
		Chronic kidney diseases	0 (0)		0 (0)		0 (0)	—
		Diabetes	16 (16)		6 (13.3)		10 (18.2)	.51
		Any of above	46 (46)		23 (51.1)		23 (41.8)	.35
	**History of confirmed SARS-Cov-2 infection**							
		No	16 (16)		3 (6.7)		13 (23.6)	—
		Yes	84 (84)		42 (93.3)		42 (76.4)	.02
**Vaccination history**								
	**Receiving seasonal influenza vaccination in the 2023/2024 flu season (October 2023 to September 2024)**							
		No	58 (58)		29 (64.4)		29 (52.7)	—
		Yes	42 (42)		16 (35.6)		26 (47.3)	.24
	**Number of doses of COVID-19 vaccination**							
		≥3	78 (78)		34 (75.6)		44 (80)	—
		2	12 (12)		7 (15.6)		5 (9.1)	—
		0-1	10 (10)		4 (8.9)		6 (10.9)	.60

^a^SIV: seasonal influenza vaccination.

^b^Not applicable. We used chi-square tests to compare the between-group difference in categorical data. Only one *P* value will be obtained from one chi-square test, regardless of the number of categories of this variable.

^c^CSSA: Comprehensive Social Security Assistance Scheme, which provides a safety net for Hong Kong residents who cannot support themselves financially to meet their basic needs.

### Actual Uptake, Behavioral Intention and Attitudes Related to SIV

The number of participants who reported a SIV uptake was 6 (13.3%) at T1 and 15 (33%) in the SIV-ChatGPT group. At T2, the SIV uptake rate was higher in the SIV-ChatGPT group than the comparison group (15/45, 33% vs 8/56, 14.3%; OR 2.94, 95% CI 1.11-7.77, *P*=.03; adjusted OR 2.72, 95% CI 1.01-7.35, *P*=.05). All participants were able to provide receipts to validate their SIV uptake. In the SIV-ChatGPT group, 40.5% (15/37) of participants who used SIV-ChatGPT at least once reported a SIV uptake at T2, which was significantly higher than nonusers (0/8, 0%; *P*=.04).

Among participants in the SIV-ChatGPT group, the proportion of participants with an intention to receive SIV was significantly higher at T1 (30/45, 66.7% vs 18/45, 40%; *P*<.001). However, there was no change in the item response or scale scores related to attitudes toward SIV when comparing the levels measured at T1 with T0 (Table 2).

**Table 2:** Changes in attitudes toward seasonal influenza vaccination (SIV) among participants in the SIV-ChatGPT group.

			T0 (n=45), n (%)		T1 (n=45), n (%)		*P* values
Behavioral intention to receive SIV, yes^a^			18 (40)		30 (66.7)		<.001
**Perceived susceptibility to seasonal influenza (high/very high)**							
	If you do not receive SIV how high is your chance of having seasonal influenza in the incoming flu season	13 (28.9)		14 (31.1)		>.99	
**Perceived severity of seasonal influenza (high/very high)**							
	If you do not receive SIV, how high is your chance of having severe illness (eg, bronchitis, pneumonia, brain lesions, or death) due to seasonal influenza	17 (37.8)		21 (46.7)		.39	
	If you do not receive SIV, how high is your chance of having co-infection of seasonal influenza and COVID-19	17 (37.8)		24 (53.3)		.07	
	If you get seasonal influenza, the older you are, the more likely you are to become seriously ill	37 (82.2)		35 (77.8)		.75	
**Perceived benefit of SIV (agree)**							
	SIV is highly effective in protecting you from seasonal influenza	30 (66.7)		31 (68.9)		>.99	
	SIV is highly effective in preventing severe consequences of seasonal influenza	34 (75.6)		38 (84.4)		.34	
	SIV is highly effective in protecting your family members from seasonal influenza	25 (55.6)		24 (53.3)		>.99	
	Perceived Benefit Scale, mean (SD)	7.5 (1.4)		7.6 (1.6)		.84	
**Perceived barrier to receive SIV (agree)**							
	SIV has severe side effects	6 (13.3)		10 (22.2)		.29	
	Item score, mean (SD)	1.8 (0.7)		1.7 (0.8)		.85	
	SIV is too expensive for you	5 (11.1)		4 (8.9)		>.99	
	Item score, mean (SD)	1.4 (0.7)		1.5 (0.7)		.62	
	It is inconvenient for you to receive SIV	3 (6.7)		3 (6.7)		>.99	
	Item score, mean (SD)	1.3 (0.6)		1.2 (0.5)		.23	
	Your health conditions are not suitable for SIV	5 (11.1)		7 (15.6)		.69	
	Item score, mean (SD)	1.4 (0.7)		1.3 (0.7)		.42	
**Perceived self-efficacy (agree)**							
	You are confident to receive SIV if you want to	39 (86.7)		41 (91.1)		.69	
	Taking up SIV is easy for you	37 (82.2)		41 (91.1)		.29	
	Perceived Self-efficacy Scale, mean (SD)	5.5 (0.9)		5.7 (0.9)		.54	

^a^Six participants who had received SIV at T1 were considered to have the intention to receive SIV in the analysis.

### Usability and Engagement With SIV-ChatGPT

During the intervention period, 37 participants (82.2%) used SIV-ChatGPT at least once (once: 26/45, 57.8%; twice: 7/45, 15.6%; and 3 times: 4/45, 8.9%). Among the users (n=37), over 70% agreed that the app was easy to use (26/37, 70.2%), the various functions in the app were well integrated (28/37, 75.6%), and they were confident to use the app (26/37, 70.2%). About 30% of the users perceived the app was unnecessarily complex (12/37, 32.4%), believed that they need support from a technical person to be able to use the app (12/37, 32.4%), found the app very cumbersome to use (10/37, 27.0%), and thought that they needed to learn a lot before they could get going with the app (11/37, 29.7%). The mean score of the SUS was 67.1 (SD 14.9). Regarding behavioral engagement with SIV-ChatGPT, about 70% perceived the app was a part of their daily routine (24/37, 64.8%), easy to use (26/37, 70.2%), and able to use it as often as they needed (31/37, 83.7%). In addition, 40.5% (n=15) of the users believed that the app motivates them to receive SIV, and 83.7% (n=31) agreed that the app helped them to get more insight into SIV. Furthermore, about half of the users agreed with the items reflecting affective engagement with the app (Table 3).

**Table 3:** Perceived usability and subjective engagement with seasonal influenza vaccination (SIV)-ChatGPT (among 37 SIV-ChatGPT users).

Category				Values, n (%)
**Perceived usability of the app (agree/strongly agree)**				
	I think that I would like to use the app frequently			22 (59.4)
	I found the app unnecessarily complex			12 (32.4)
	I thought the app was easy to use			26 (70.2)
	I think I would need the support from a technical person to be able to use this app			12 (32.4)
	I found the various functions in this app were well integrated			28 (75.6)
	I thought there was too much inconsistency in this app			4 (10.8)
	I would imagine that most people would learn to use this app very quickly			24 (64.8)
	I found the app very cumbersome to use			10 (27)
	I felt very confident using the app			26 (70.2)
	I needed to learn a lot of things before I could get going with this app			11 (29.7)
**Subjective engagement with the app**				
	**Behavioral engagement (strongly agree/agree)**			
		The app is a part of my daily routine	24 (64.8)	
		The app is easy to use	26 (70.2)	
		You are able to use the app as often as you needed	31 (83.7)	
	**Cognitive engagement (strongly agree/agree)**			
		The app makes it easier for me to work on my goal	29 (78.3)	
		The app motivates me to receive SIV	15 (40.5)	
		The app helps me to get more insight into SIV	31 (83.7)	
	**Affective engagement (strongly agree/agree)**			
		You enjoyed using the app	21 (56.7)	
		I enjoyed seeing the progress I made when using the app	21 (56.7)	
		The app fits me as a person	19 (51.3)	

### Qualitative Feedback of SIV-ChatGPT and Sample Quotes

We identified two themes related to the strengths of the app: (1) able to obtain comprehensive information by using the app, and (2) information provided by the app is credible, useful, and easy to understand. Four other themes were related to areas that needed improvement: (1) difficulties to login the web-based app, (2) too many words in responses generated by the app, (3) font size of responses generated by the app was too small for older adults, and (4) suggestions for providing information related to other common chronic conditions in addition to SIV. Sample quotes for the above themes were listed in Table 4. We did not receive complaints about erroneous recommendations/responses from users during the study period.

**Table 4:** Qualitative feedback of seasonal influenza vaccination (SIV)-ChatGPT and sample quotes.

Themes	Sample quotes
Able to obtain comprehensive information by using the app	“My knowledge (related to seasonal influenza) increased after using the Application” [Informant 12, female, aged 67 years, used SIV-ChatGPT twice] “I can learn more about seasonal influenza. The information is comprehensive” [Informant 24, female, aged 67 years, used SIV-ChatGPT for 3 times]
Information provided by the app is credible, useful and easy to understand	“Information provided by the Application was identical to those in the materials provided by local hospitals” [Informant 43, female, aged 74 years, used SIV-ChatGPT once] “The Application has done well in promoting older adults to receive SIV” [Informant 4, aged 71 years, used SIV-ChatGPT once] “The Application can explain who needs to receive SIV in simple terms” [Informant 15, aged 69 years, used SIV-ChatGPT once]
Difficulties to login the app	“I cannot login the Application. I have to ask my husband to help me” [Informant 16, female, aged 67 years, used SIV-ChatGPT once] “The login was too difficult for me. I tried but failed. Then I gave up” [Informant 28, female, aged 67 years, did not use SIV-ChatGPT] “I had to ask my children to help me login. They are not around. So I did not use the Application” [Informant 7, male, aged 73 years, did not use SIV-ChatGPT]
Too many words in responses generated by the app	“Better to reduce the number of words (in SIV-ChatGPT-generated responses). Too many words now. Adding figures will make the contents more clear” [Informant 3, female, aged 71 years, used SIV-ChatGPT twice] “Too many words. Need to spend quite some time to read it through” [Informant 14, male, aged 69 years, used SIV-ChatGPT once]
Font size of responses generated by the app was too small	“The communication is smooth. Larger font size will be better for older adults” [Informant 42, male, aged 67 years, used SIV-ChatGPT once]
Providing information related to other common chronic conditions in addition to SIV	“Better to cover information of other common chronic conditions” [Informant 6, female, aged 70 years, used SIV-ChatGPT once]

## Discussion

### Principal Findings

This was one of the first studies that examined the effectiveness, usability, and feasibility of ChatGPT in increasing vaccination uptake among older adults. Other strengths of this study included the use of RAG and a comprehensive QA database to increase the accuracy of ChatGPT-generated responses, having independent experts to validate the accuracy of the response, and using a random and population-based sample for evaluation. Therefore, our findings contributed to the development of new methods to increase SIV uptake among adults aged 65 years or older.

In line with our hypothesis, the validated SIV uptake rate in the SIV-ChatGPT group was significantly higher than that of the comparison group (15/45, 33.3% vs 8/55, 14.3%; *P*=.048). Within the SIV-ChatGPT group, those who had used the ChatGPT at least once reported significantly higher SIV uptake than nonusers (n=15, 40.6% vs n=0, 0%; *P*=.04). These findings provided preliminary evidence supporting the effectiveness of SIV-ChatGPT in increasing SIV uptake. Our project started 2 months after the rollout of the governmental SIV program in October 2024. Statistics showed that the majority of the older adults in Hong Kong who planned to receive SIV for the approaching flu season would receive the vaccine 1-2 months after the program rollout [6]. It was possible that our sample represented a group of older adults who were more hesitant to receive SIV. Meta-analysis indicated that providing adequate information could significantly increase SIV uptake compared with no intervention [7]. Reflected by users’ qualitative feedback, the ability to generate comprehensive and credible responses that were easy to understand might contribute to the effectiveness of SIV-ChatGPT. A rule-based chatbot was developed to promote SIV in the same population between 2021 and 2022 [20]. The chatbot operated based on predetermined rules but could not address users’ questions in real-time. Half of the users of this chatbot received an SIV 6 months after completion of the interventions. Such SIV uptake rate was slightly higher than that observed in the SIV-ChatGPT group over a shorter follow-up period (2 months after completion of the interventions). In the future, a hybrid chatbot that combines the rule-based chatbot and SIV-ChatGPT should be considered. The rule-based part ensures carefully designed interventions are delivered as intended, while the ChatGPT part enables the chatbot to better understand users’ questions and to generate real-time responses. Such a combination may improve effectiveness and user experience.

The usability of SIV-ChatGPT was supported by the mean SUS score of 67.1 out of 100, which was close to the cut-off of “good” (a score of 71.4) [29]. Over 80% of the older adults used SIV-ChatGPT at least once. The extent of use was slightly higher than the level for a rule-based chatbot promoting SIV uptake in the same age group (77.3%) [20]. In addition, the levels of subjective behavioral and cognitive engagement with SIV-ChatGPT were relatively high. However, the usability and acceptability data also suggested a need for improvement. About 30% of the users found SIV-ChatGPT complex and cumbersome to use, and they needed to learn a lot of things or sought help from other people to use it. These issues might negatively affect the level of affective engagement with SIV-ChatGPT, which was only moderate in this study (about 50% of users agreed with relevant items). Qualitative feedback from the participants suggested possible causes of these issues and ways for improvement. First, we assigned a unique username and password (a combination of both letters and numbers) to each participant in the SIV-ChatGPT group to facilitate user monitoring and to avoid contamination. However, this approach created unexpected difficulties for older adults to log in to the web-based app. Some participants reported difficulties entering the username or password on their mobile devices; a few of them gave up using SIV-ChatGPT due to such difficulties. In the future, older adults should be able to access SIV-ChatGPT without any login procedures. Second, some users commented that there were too many words in the ChatGPT-generated responses, and the font size was too small for them. They wished the responses could involve figures and videos to present complex health communication messages. It is feasible to provide a choice of font size to cater to the needs of users of different age groups in the future. However, human-LLM interactions currently mainly rely on text interaction, which may be a constraint for ChatGPT and other LLM chatbots. Moreover, users wished SIV-ChatGPT could cover information of other chronic conditions. Therefore, the QA databases should be expanded to cover common chronic conditions among local older adults.

### Limitations

This study has some limitations. First, we were not able to use a randomized controlled trial to evaluate SIV-ChatGPT due to the constraints of funding and resources. The recruitment and data collection methods of the comparison group were identical to the SIV-ChatGPT group, and the baseline characteristics were similar between groups. However, the quasi-experimental design could not control for all factors that affect the internal validity, and could only provide supportive evidence about the effectiveness. Second, the sample size was relatively small, which precludes the examination of SIV-ChatGPT’s effectiveness in different subgroups. The sample size of the SIV-ChatGPT group was comparable to previous studies evaluating the usability and acceptability of chatbots [30,31]. Third, we were not able to observe the long-term effects of SIV-ChatGPT due to the short follow-up duration. Fourth, the intervention was limited to adults aged 65 years or older who had access to smartphones. In Hong Kong, 96.4% of residents aged 60 years or older owned a smartphone in 2023, and WhatsApp is the most commonly used instant messaging app in the city [32]. It is expected that very few older adults in Hong Kong do not have access to a smartphone or WhatsApp. Fifth, people aged 75 years or older were undersampled in this study [33]. The income, education level, and presence of chronic conditions among our participants in the SIV-ChatGPT group and the comparison group were similar to those of a more representative sample in a recent random telephone survey [19]. Since older age was associated with poorer digital literacy, the effectiveness, usability, and acceptability of SIV-ChatGPT might be overestimated in this study [34]. Sixth, 24% (n=14) of older adults, of whom we were unable to collect their information, were assessed for eligibility but refused to use SIV-ChatGPT. It was possible that older adults who had a higher motivation to receive an SIV would be more willing to join this study. Such self-selection bias would also lead to an overestimation of the effectiveness, usability, and acceptability of SIV-ChatGPT. Moreover, behavioral intention, attitudes, usability, and subjective engagement were self-reported and could not be validated. Participants might over-report these outcomes due to social desirability. Furthermore, this study was conducted in Hong Kong. Due to the difference in health care system, vaccination delivery models, digital literacy, and cultural factors, the findings might not be applicable to other settings, especially in low-and-middle-income countries (LMICs). ChatGPT is usually operated through smartphones, which presents challenges to its accessibility in LMICs. Moreover, ChatGPT may require some digital literacy to operate, which could be challenging for individuals of low socioeconomic status. Future studies should explore the feasibility of the SIV-ChatGPT in LMICs. Nevertheless, the findings must be considered preliminary and hypothesis testing. Last but not least, the selection of the intervention period (1 month) was mainly based on the scope of funding and project period.

### Conclusions

In sum, SIV-ChatGPT in the format of a web-based app was feasible and acceptable and demonstrated preliminary effectiveness in increasing SIV uptake among people aged 65 years or older in Hong Kong. This study also provided implications to improve the performance of SIV-ChatGPT. A full-powered randomized controlled trial should be considered in the future to evaluate its efficacy. SIV-ChatGPT, if proven to be efficacious by the randomized controlled trial, will provide a sustainable approach to supplement the existing methods of SIV information dissemination (mass media, webpage, posters). SIV-ChatGPT could be adopted by governmental and nongovernmental organizations serving older adults, and governmental vaccination webpages to enhance the effectiveness of governmental SIV programs.

## Abbreviations

LLM: large-language model
LMIC: low-and-middle-income country
OR: odds ratio
QA: question-answer
RAG: retrieval-augmented generation
SIV: seasonal influenza vaccination
SUS: System Usability Scale
TWEETS: Twente Engagement with eHealth Technologies Scale

## Data Availability

The datasets generated or analyzed during this study are not publicly available because they contained information about sensitive personal characteristics and behaviors, but are available from the corresponding author on reasonable request.
